# Arterial tortuosity syndrome: A rare entity

**DOI:** 10.4103/0974-2069.41060

**Published:** 2008

**Authors:** Ashutosh Marwah, Sejal Shah, P.V. Suresh, Sunita Maheshwari

**Affiliations:** Department of Pediatric Cardiology, Narayana Hrudayalaya Institute of Cardiac Sciences, Bangalore, India

**Keywords:** Arterial tortuosity syndrome, tortuous pulmonary arteries

## Abstract

We present a 5 month old baby who was referred for an incidental detection of a murmur and was found to have tortuous pulmonary arteries with multiple peripheral pulmonary stenoses and bilateral inguinal hernia pointing towards the diagnosis of arterial tortuosity syndrome.

## INTRODUCTION

Arterial tortuosity syndrome is a rare connective tissue disorder of unknown etiology. The most prominent features of this syndrome are lengthening of major arteries and aneurysm formation. Since its first description in 1969 by Less *et al*,[1] there have also been few reports of similar patients born out of consanguinous marriages. We present a case of this relatively uncommon syndrome as yet not reported from the Indian subcontinent.

## CASE REPORT

A five month-old boy was referred for evaluation after his pediatrician detected a murmur. He was born at term and there was no history of consanguinity. He had history of fast breathing and poor weight gain for the last two months. Clinical examination revealed normal facies and skin texture, a pulse rate of 96/min and oxygen saturation on room air of 88%. Cardiac apex impulse was in the 5th left intercostal space. The first and second heart sounds were normal, with systolic murmur heard all over the chest. Liver was palpable 2 cm below the costal margin. In addition he had bilateral inguinal hernias.

The X-ray chest revealed cardiomegaly with normal pulmonary vascularity. The 12 lead ECG showed a sinus rhythm with right axis deviation and right ventricular hypertrophy. A cross-sectional echocardiography and Doppler examintation revealed normal segmental subset. There was a right to left shunt across a patent foramen ovale. There was no ventricular septal defect or aortopulmonary shunt. The right ventricle was dilated and had mildly reduced contractility. There was moderate tricuspid regurgitation with a jet velocity of > 4m/sec. There was no valvar pulmonary stenosis but the branch pulmonary arteries appeared tortuous and there was suspicion of peripheral stenoses.

**Figure 1:**
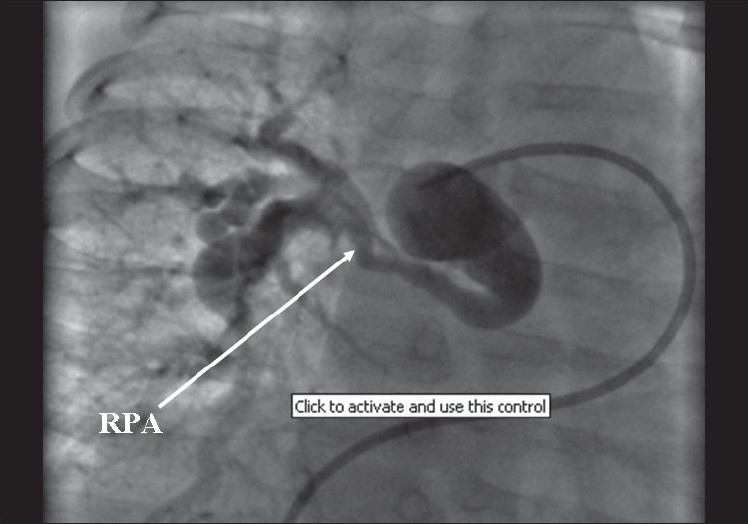
Pulmonary artery angiogram in AP view showing tortuous right pulmonary artery with peripheral stenoses. RPA= right pulmonary artery

**Figure 2:**
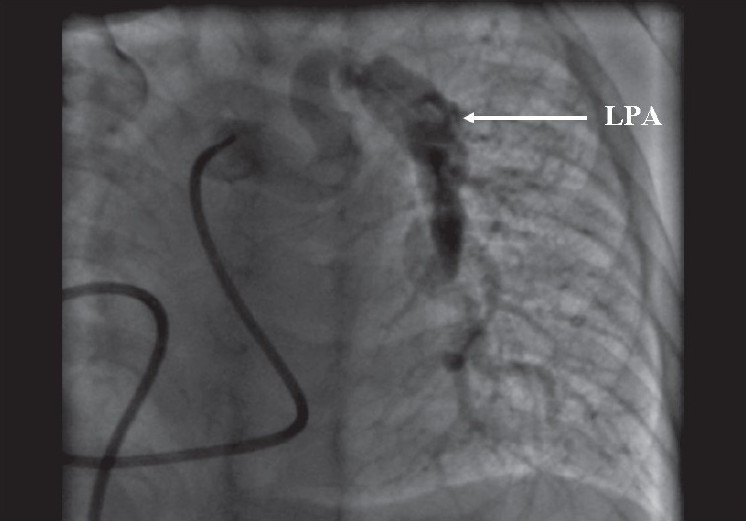
Pulmonary artery angiogram in AP view showing tortuous left pulmonary artery with stenosis. LPA= left pulmonary artery

**Figure 3:**
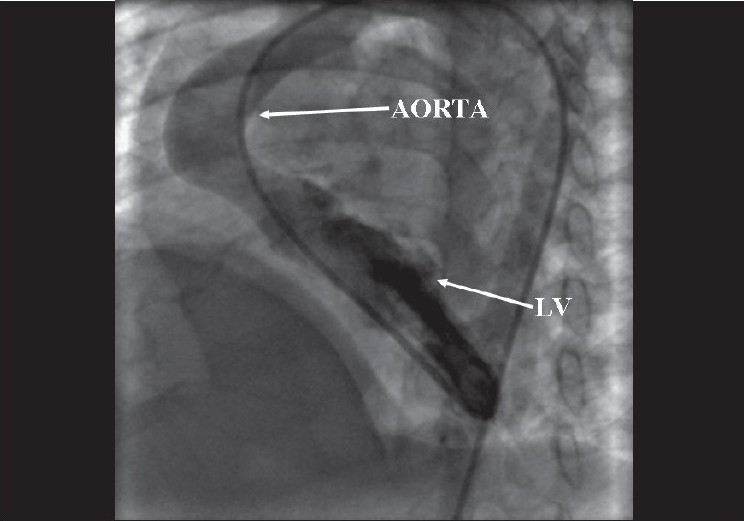
Left ventricular angiogram in LAO view showing elongated aortic root and aortic arch. LV= left ventricle

In view of these findings, cardiac catheterization was performed to confirm the hemodynamic data and to delineate the pulmonary artery anatomy. Pressure and oxygen saturation data is given in Table 1. Angiography revealed tortuous pulmonary arteries with multiple areas of peripheral stenoses [Figures 1 and 2]. The interventricular septum was intact. The aortic root and arch appeared elongated [Figure 3]. There was no arterial duct but the subclavian artery also showed a tortuous course. As no suitable therapeutic option could be offered, the patient was discharged on medical follow-up.

**Table 1:** The pressure data and oxygen saturation on cardiac catheterization

Site	Pressure	Saturation%
Right atrium	Mean=7	67
Right ventricle	110/15	72
Main pulmonary artery	100/30	74
Aorta	90/60, m=70	88
Left ventricle	90/10	

## DISCUSSION

Arterial tortuosity syndrome is an uncommon connective tissue disorder of unknown etiology. Various authors have reported occurrence of sporadic cases in the world literature.[2–4] These patients have a variable degree of skin hyperextensibility, hypermobility of small and large joints and peculiar facial features consisting of epicanthic folds, flat saggy cheeks, elongated facies and micrognathia.[2–4] In addition, there is a variable degree of elongation and tortuosity of systemic and pulmonary vessels along with formation of aneurysms and multiple areas of stenoses as was seen in the present case. The histological findings of arterial changes with disruption of elastic fibers of the media and fragmentation of the internal elastic membrane have been described. The arterial tortuosity syndrome (ATS) gene has been mapped to chromosome 20q13.[5]

In a series reported by Wahab *et al*,[6] the combination of an elongated aortic arch and tortuous brachiocephalic arteries was seen in 30 patients (94%), aneurysm of the ascending aorta in three patients (9.4%), bifid pulmonary artery in 27 patients (84%) and multiple severe peripheral stenosis of the right and/or left pulmonary artery in seven patients (22%). A prominent aortic knuckle was observed on the chest roentgenograms in 30 patients (94%), inguinal hernia in 11 (34%), diaphragmatic hernia and/or hiatus hernia in 7 (22%); and laryngo-tracheomalacia in 2 (6.3%). Generalized muscle hypotonia was found in 15 neonates (47%). Parental consanguinity was seen in all the patients and was traced to a common ancestor from a large Bedouin tribe in Qatar.

Although our patient did not have a history of consanguinity and lacked all the physical features characteristic of this syndrome, his pulmonary artery angiograms were typical of this disorder. He also had an elongated aortic arch, tortuosity of subclavian artery and bilateral inguinal hernia as additional pointers to the diagnosis. As far as we are aware, this is the only published report of this uncommon syndrome from the Indian subcontinent. In cases where there is unexplained high right ventricular pressure with features suggestive of peripheral pulmonary artery stenosis, a cardiac catheterization can be useful in picking up this rare disorder of the pulmonary artery. In view of the generalized pulmonary vascular involvement, therapeutic options were limited in our patient as has been the case with the majority of patients reported in the literature.
